# Vitamin D-metabolic enzymes and related molecules: Expression at the maternal-conceptus interface and the role of vitamin D in endometrial gene expression in pigs

**DOI:** 10.1371/journal.pone.0187221

**Published:** 2017-10-31

**Authors:** Hwanhee Jang, Yohan Choi, Inkyu Yoo, Jisoo Han, Jin Su Hong, Yoo Yong Kim, Hakhyun Ka

**Affiliations:** 1 Department of Biological Science and Technology, Yonsei University, Wonju, Republic of Korea; 2 Department of Agricultural Biotechnology, Seoul National University, Seoul, Republic of Korea; University of Alabama at Birmingham, UNITED STATES

## Abstract

Vitamin D is a secosteroid hormone with many varied functions including regulation of blood calcium levels, cell proliferation, immunity, and reproduction in mammals. Vitamin D is activated by 25-hydroxylase (CYP2R1) and 1-alpha-hydroxylase (CYP27B1) and is degraded by 24-hydroxylase (CYP24A1). Vitamin D is transported by vitamin D-binding protein (group-specific component, GC) through the bloodstream and regulates cellular actions by binding to vitamin D receptor (VDR). In this study, we determined the expression and regulation of vitamin D-related molecules and the role of vitamin D at the maternal-conceptus interface in pigs. Vitamin D-metabolizing enzymes *CYP2R1*, *CYP27B1*, and *CYP24A1*, vitamin D binding protein *GC*, and vitamin D receptor *VDR* were expressed in the endometrium in a pregnancy stage-specific manner as well as in conceptus and chorioallantoic tissues during pregnancy. VDR protein was localized to endometrial and trophoblastic cells. Concentrations of calcitriol, the active form of vitamin D, in the endometrial tissues were higher during early pregnancy than in mid- to late pregnancy, while plasma concentrations of calcitriol were highest during late pregnancy. Furthermore, calcitriol affected the expression of several genes related to conceptus implantation, vitamin D metabolism, calcium ion regulation, PG metabolism, and calcium-binding proteins in endometrial tissue explants. These results show that *CYP2R1*, *CYP27B1*, *CYP24A1*, *GC*, and *VDR* were expressed at the maternal-conceptus interface, endometrial calcitriol levels were regulated during pregnancy, and calcitriol modulated the expression of endometrial genes, suggesting that calcitriol may play an important role in the establishment and maintenance of pregnancy by regulating endometrial function in pigs.

## Introduction

Appropriate interactions between the developing conceptus (embryo/fetus and associated extraembryonic membranes) and the maternal endometrium are essential for the establishment and maintenance of pregnancy and are tightly regulated by many factors including steroid hormones, prostaglandins (PGs), cytokines, enzymes, and ions [1, 2]. During the implantation period in pigs, conceptuses change morphology from spherical to filamentous forms and secrete estrogen that functions as the maternal recognition signal of pregnancy [2]. The conceptus-derived estrogen redirects PGF_2α_ secretion from the uterine vasculature to the uterine lumen for corpora lutea (CL) maintenance and induces the expression of many endometrial genes including aldo-keto reductase 1B1 (*AKR1B1*) [3], fibroblast growth factor 7 (*FGF7*) [4], lysophosphatidic acid receptor 3 (*LPAR3*) [5]), S100 calcium binding protein G (*S100G*) [6], secreted phosphoprotein 1 (*SPP1*) [7], signal transducer and activator of transcription 1 (*STAT1*) [8], stanniocalcin 1 (*STC1*) [9], and transient receptor potential cation channel subfamily V member 6 (*TRPV6*) [10]. Estrogen also increases endometrial secretion of calcium, total protein, PGE_2_, and PGF_2α_ into the uterine lumen at the time of conceptus implantation [11]. The implanting porcine conceptuses also produce cytokines, interleukin-1β2 (IL1β2), interferon-δ (IFND), and IFN-γ (IFNG), which activate many immune regulatory molecules in the endometrium [2].

In pigs, many studies have been performed to determine the roles of factors affecting the establishment and maintenance of pregnancy, but the function of vitamin D at the maternal-conceptus interface is not well understood. Vitamin D is a well-known secosteroid hormone that plays a critical role in bone metabolism and calcium homeostasis [12,13]. Vitamin D increases blood calcium levels through absorption of calcium in the gastrointestinal tract, reabsorption of calcium in the kidney, and release of calcium release from the bone [14]. In addition, vitamin D is involved in regulation of cell proliferation, immunity, and reproduction in mammals [15–17]. In humans and animals [13,14], cholecalciferol, a biologically inactive form of vitamin D, is obtained from foods or is synthesized in the skin by exposure to ultraviolet B light and then undergoes two hydroxylation events by hydroxylases to form the active form of vitamin D, 1,25-dihydroxyvitamin D_3_ [calcitriol, 1,25(OH)_2_D_3_]. Cholecalciferol is circulated bound with vitamin D binding protein (also known as group-specific component; GC) or albumin in blood and is hydroxylated to calcifediol [25(OH)D_3_], the major circulating form, by CYP2R1 (25-hydroxylase) in the liver [14]. Calcifediol is hydroxylated to calcitriol [1,25(OH)_2_D_3_] by CYP27B1 (1-alpha-hydroxylase) in the kidney, placenta, and other tissues [15,18]. Calcitriol binds to the vitamin D receptor (VDR), which is a member of the nuclear receptor family of transcription factors and mediates most of the physiological actions of vitamin D [13]. In tissues, calcitriol is changed to its inactive form, calcitroic acid, by CYP24A1 (24-hydroxylase) and is excreted [18]. CYP24A1 also hydroxylates calcifediol to 24*R*-hydroxycalcidiol [24,25(OH)_2_D_3_] in the kidney (18). In many tissues, the actions of calcitriol are mediated by genomic and/or non-genomic signaling pathways [19]. In the genomic signaling pathway, calcitriol binds to VDR with a partner receptor, retinoid X receptor, to regulate transcription of vitamin D target genes, whereas calcitriol binds to VDR associated with caveolae to activate various intracellular signaling cascades in the non-genomic pathway [19].

Expression of vitamin D metabolizing enzymes and production of vitamin D at the maternal-fetal interface have been shown in humans and rodents, suggesting that vitamin D plays important roles in the establishment and maintenance of pregnancy [14, 20–22]). *CYP2R1* is expressed in human placentas [23] and uterine endometrial cells with greater abundance during the secretory phase than during the proliferative phase [24]. Placental expression of *CYP27B1* mRNA and protein is positively correlated with levels of maternal vitamin D in humans [25], and placental expression of *CYP24A1* is decreased during pregnancy due to methylation of the promoter region of the *CYP24A1* gene in humans and rodents [26]. Levels of *VDR* expression increase in placenta and decidua as pregnancy progresses and are higher in the endometrium during pregnancy than in the estrous cycle in mice [27]. In addition, it has been shown that human endometrial decidua and placental tissues synthesize calcitriol and calcifediol in vitro [28–31], indicating the presence of independent vitamin D metabolism activity in the feto-maternal unit. However, expression of *CYP2R1*, *CYP27B1*, *CYP24A1*, *GC*, and *VDR* in the endometrium during the estrous cycle and pregnancy has not been fully studied in domestic animal species.

Mice lacking *Vdr* are infertile, indicating a critical role of vitamin D action in reproduction [32]. In addition to classical actions of vitamin D on calcium homeostasis, it is well known that vitamin D also has immunomodulatory, anti-inflammatory, and anti-proliferative properties [22, 33, 34]. Although many studies indicate that vitamin D plays important roles in the establishment and maintenance of pregnancy, and the roles of vitamin D at the maternal-fetal interface have been investigated in humans and rodents, the expression of vitamin D system and function of vitamin D at the maternal-conceptus interface have not been investigated in pigs.

Therefore, to initiate a study on the role of vitamin D at the maternal-conceptus interface during pregnancy in pigs, we determined: 1) expression of vitamin D hydroxylases (*CYP2R1*, *CYP24A1*, and *CYP27B1*), *DC*, and *VDR* in the endometrium during the estrous cycle and pregnancy, as well as in the early stage conceptus and chorioallantoic membrane during pregnancy; 2) localization of *CYP2R1*, *CYP24A1*, and *CYP27B1*, *DC*, and *VDR* mRNAs in the endometrium; 3) concentration of calcitriol in plasma, endometrium, and uterine lumen during the estrous cycle and pregnancy; and 4) effect of calcitriol on the expression of endometrial genes related to calcium homeostasis, conceptus implantation, and immune responses.

## Materials and methods

### Animals and tissue collection

All experimental procedures involving animals were conducted in accordance with the Guide for Care and Use of Research Animals in Teaching and Research and were approved by the Institutional Animal Care and Use Committee of Yonsei University (Approval No. YWC-P120). Sexually mature crossbred female pigs were assigned randomly to either the cyclic or pregnant status. The reproductive tracts of gilts were obtained immediately after slaughter on either Days 12 or 15 of the estrous cycle or on Days 12, 15, 30, 60, 90 or 114 of pregnancy (n = 4–6 gilts/day/status). Pregnancy was confirmed by the presence of apparently normal filamentous conceptuses in uterine flushings on Days 12 and 15 and the presence of embryos and placenta on the later days of pregnancy. Uterine flushings on Days 12 and 15 post-estrus (n = 3–6/day/status) were obtained by introducing and recovering 50 ml phosphate buffered saline (PBS, pH 7.4) from the uterus (25 ml/uterine horn). Conceptus tissues on Days 12 and 15 of pregnancy were obtained from uterine flushings, and chorioallantoic tissues were obtained on Days 30, 60, 90, and 114 of pregnancy (n = 3–4/day).

The endometrium, dissected free of myometrium, was collected from the middle portion of each uterine horn, snap-frozen in liquid nitrogen, and stored at -80°C for RNA extraction. For *in situ* hybridization analysis and immunohistochemistry, cross-sections of endometrium were fixed in 4% paraformaldehyde in PBS (pH 7.4) for 24 h and then embedded in paraffin as previously described [5].

### Total RNA extraction and cloning of *CYP2R1*, *CYP27B1*, *CYP24A1*, *GC*, and *VDR* cDNAs

Total RNA was extracted from endometrial, chorioallantoic, and conceptus tissues using TRIzol reagent (Invitrogen, Carlsbad, CA) according to the manufacturer’s recommendation. The quantity of RNA was assessed spectrophotometrically, and the integrity of the RNA was validated following electrophoresis using a 1% agarose gel.

Total RNA (4 μg) was isolated from endometrial and chorioallantoic tissues and conceptus tissues, treated with DNase I (Promega, Madison, WI), and reverse transcribed using SuperScript II Reverse Transcriptase (Invitrogen) to obtain cDNAs. The cDNA templates were diluted 1:4 with nuclease-free water and amplified by PCR using Taq polymerase (Takara Bio, Shiga, Japan). The PCR conditions and sequences of primer pairs are listed in S1 Table. The PCR products were separated on 2% agarose gels and visualized by ethidium bromide staining. The identity of each amplified PCR product was verified by sequence analysis after cloning into the pCRII vector (Invitrogen).

### Quantitative real-time RT-PCR

To analyze transcription levels of uterine endometrial genes in the endometrium, real-time RT-PCR was performed using the SYBR Green method with the Applied Biosystems StepOnePlus System (Applied Biosystems, Foster City, CA). Complementary DNAs were synthesized from 4 μg total RNA isolated from different uterine endometrial tissues, and newly synthesized cDNAs (total volume of 21 μl) were diluted 1:4 with nuclease-free water and then used for PCR. The Power SYBR Green PCR Master Mix (Applied Biosystems) was used for PCR reactions. The final reaction volume of 20 μl included 2 μl of cDNA, 10 μl of 2X Master mix, 2 μl of each primer (100 nM), and 4 μl of dH_2_O. PCR conditions and sequences of primer pairs are listed in S1 Table. The results are reported as the expression relative to the level detected on Day 12 of the estrous cycle after normalization of the transcript amount to the endogenous porcine ribosomal protein L7 (*RPL7*) control by the 2^-ΔΔCT^ method [35].

### Non-radioactive in situ hybridization

The non-radioactive *in situ* hybridization procedure was performed as described previously [36] minor modifications. Sections (5 μm thick) were rehydrated through successive baths of xylene, 100% ethanol, 95% ethanol, diethylpyrocarbonate (DEPC)-treated water, and DEPC-treated PBS. Tissue sections were boiled in citrate buffer (pH 6.0) for 10 min. After washing in DEPC-treated PBS, tissue sections were digested using 5 μg/ml Proteinase K (Sigma, St Louis, MO) in TE (100 mM Tris-HCl, 50 mM EDTA, pH 7.5) at 37°C. After post-fixation in 4% paraformaldehyde, sections were incubated twice for 15 min each in PBS containing 0.1% active DEPC and equilibrated for 15 min in 5X saline sodium citrate (SSC). The sections were prehybridized for 2 h at 68°C in hybridization mix (50% formamide, 5X SSC, 500 μg/ml herring sperm DNA, 250 μg /ml yeast tRNA; 200 μl on each section). Sense and antisense *CYP2R1*, *CYP24A1*, *CYP27B1*, *DC*, and *VDR* riboprobes were generated using partial cDNAs cloned into pCRII vectors by linearizing with appropriate restriction enzymes and labeling with digoxigenin (DIG)-UTP using a DIG RNA Labeling kit (Roche, Indianapolis, IN). The probes were denatured for 5 min at 80°C and added to the hybridization mix, and the hybridization reaction was carried out at 68°C overnight. Prehybridization and hybridization reactions were performed in a box saturated with a 5X SSC—50% formamide solution to avoid evaporation, and no coverslips were used. After hybridization, sections were washed for 30 min in 2X SSC at room temperature, 1 h in 2X SSC at 65°C, and 1 h in 0.1X SSC at 65°C. Probes bound to the section were detected immunologically using sheep anti-DIG Fab fragments covalently coupled to alkaline phosphatase and nitro blue tetrazolium chloride/5-bromo-4-chloro-3-indolyl phosphate (toluidine salt) as the chromogenic substrate, according to the manufacturer’s protocol (Roche).

### Explant culture

The endometrial tissues from pigs on Day 12 of the estrous cycle were dissected from the myometrium and placed into warm phenol Dulbecco modified Eagle medium/F-12 (DMEM/F-12) culture medium (Sigma, St. Louis, MO) containing penicillin G (100 IU/ml) and streptomycin (0.1 mg/ml) as described previously (5) with some modifications. The endometria were minced with scalpel blades into small pieces (2–3 mm^3^), and aliquots of 500 mg were placed into T25 flasks with serum-free modified DMEM/F-12 containing 10 μg/ml insulin (Sigma), 10 ng/ml transferrin (Sigma), and 10 ng/ml hydrocortisone (Sigma). Endometrial explants were cultured immediately after mincing in the presence of 0, 2, 20, or 200 nM calcitriol (Enzo Life Sciences, Miami, FL) with E_2_ (10 ng/ml; Sigma) and P_4_ (30 ng/ml; Sigma) for 24 h with rocking in an atmosphere of 5% carbon dioxide in air at 37°C. Explant tissues were then harvested, and total RNA was extracted for real-time RT-PCR analysis to determine expression levels of endometrial genes. These experiments were conducted using endometria from three gilts, and treatments were performed in triplicate using endometrial tissues obtained from each gilt.

### Immunohistochemistry

To determine the type(s) of cells expressing VDR in the porcine endometrium, sections were immunostained. Sections (5 μm thick) were deparaffinized and rehydrated in an alcohol gradient. Tissue sections were washed with PBS with 0.1% (v/v) Tween-20 (PBST) and endogenous peroxidase activity was blocked with 0.5% (v/v) H_2_O_2_ in methanol for 30 min. Tissue sections were then blocked with 10% normal goat serum for 30 min at room temperature. Rabbit polyclonal anti-VDR antibody (2 μg/ml; Cat # ab137371; Abcam, Cambridge, MA) was added, and sections were incubated overnight at 4°C in a humidified chamber. For each tissue tested, purified normal rabbit IgG was substituted for the primary antibody as a negative control. Tissue sections were washed intensively with PBST. Biotinylated goat anti-rabbit secondary antibody (1 μg/ml; Vector Laboratories, Burlingame, CA) was added, and sections were incubated for 1 h at room temperature. Following washes with PBST, a streptavidin peroxidase conjugate (Invitrogen) was added to the tissue sections, which were then incubated for 10 min at room temperature. The sections were washed with PBST, and aminoethyl carbazole substrate (Invitrogen) was added to the tissue sections, which were then incubated for 20 min at room temperature. The tissue sections were washed in water and coverslipped without counterstaining.

### Analysis of calcitriol levels in plasma, uterine endometrial tissues, and uterine flushings

Blood samples from the jugular vein were collected into ethylenediaminetetraacetic acid-treated tubes (BD, Franklin Lakes, NJ), and plasma was obtained by centrifuging the samples at 2,000 x g for 10 min using a refrigerated centrifuge. Endometrial tissues were homogenized in lysis buffer (1% Triton X-100, 0.5% Nonidet P-40, 150 mM NaCl, 10 mM Tris, 1 mM EDTA, 0.2 mM Na_3_VO_3_, 0.2 M PMSF, and 0.5 μg/ml NaF) at a ratio of 100 mg tissue:1 ml buffer, and cellular debris was removed by centrifugation at 16,500 x g for 5 min. Levels of calcitriol in plasma blood, endometrial tissue lysates, and uterine flushings were measured using ELISA kits (NeoBioLab, Cambridge, MA), according to the manufacturer’s instructions. The concentrations of calcitriol in the endometrial tissues were standardized per total endometrial tissue weight, and the amounts of calcitriol in the uterine flushings were total recoverable amounts of calcitriol.

### Statistical analyses

Data from real-time RT-PCR and ELISA were subjected to ANOVA using the General Linear Models procedures of SAS (Cary, NC). As sources of variation, the model included day, pregnancy status (cyclic or pregnant, Days 12 and 15 post-estrus), and their interactions to evaluate levels of *CYP2R1*, *CYP27B1*, *CYP24A1*, GC, and *VDR* mRNAs in the endometrium and calcitriol levels in the endometrium and in uterine flushings. Least square regression analysis was used to evaluate data from real-time RT-PCR performed to assess the effect of the day of pregnancy (Days 12, 15, 30, 60, 90, and 114) in the endometrium and the effect of the day of pregnancy (Days 30, 60, 90, and 114) in chorioallantoic tissue for the expression of *CYP2R1*, *CYP27B1*, *CYP24A1*, GC, and *VDR*, data from ELISA to assess the effect of the day of pregnancy in plasma and endometrial tissue for calcitriol levels, and data from calcitriol dose-response studies. Data are presented as mean with SEM.

## Results

### Expression of vitamin D_3_ hydroxylases, *CYP2R1*, *CYP24A1*, and *CYP27B1*, vitamin D binding protein, *DC*, and vitamin D receptor, *VDR*, in the endometrium during the estrous cycle and pregnancy in pigs

**Fig 1:**
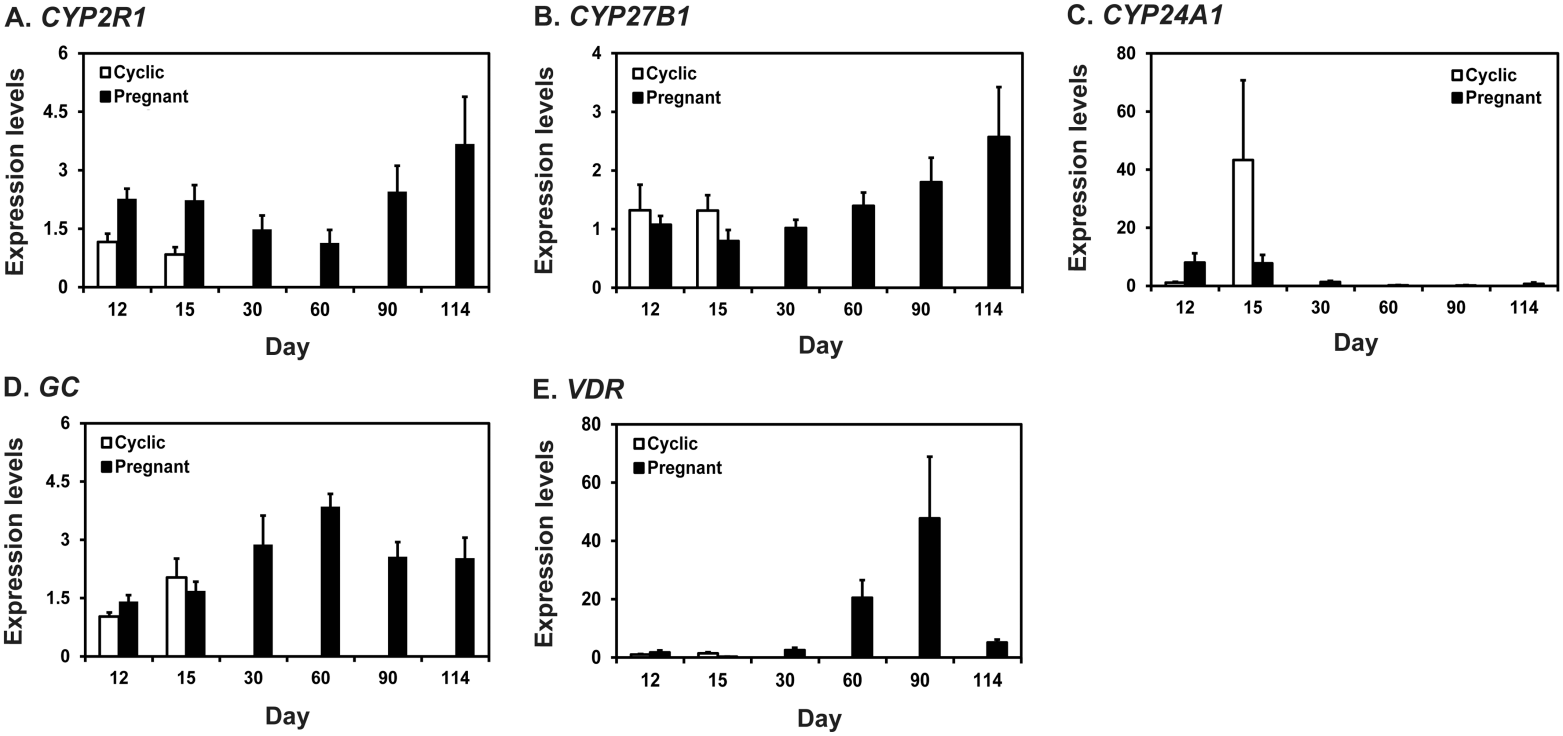
Expression of *CYP2R1* (A), *CYP27B1* (B), *CYP24A1* (C), *GC* (D), and *VDR* (E) mRNAs in the endometrium during the estrous cycle and pregnancy in pigs. Endometrial tissue samples from cyclic and pregnant gilts were analyzed by real-time RT-PCR, and data are reported as expression relative to that detected on Day 12 of the estrous cycle after normalization of the transcript amount to the endogenous *RPL7* control. Data are presented as mean with standard error.

To determine whether vitamin D hydroxylases, *CYP2R1*, *CYP24A1*, and *CYP27B1*, vitamin D binding protein, *DC*, and vitamin D receptor, *VDR*, were expressed in the endometrium in pigs, we performed real-time RT-PCR (Fig 1). Expression of *CYP2R1* mRNA was affected by pregnancy status (*P* < 0.01), but not by day and day × status interaction, on Days 12 and 15 post-estrus. Steady-state levels of *CYP2R1* mRNAs during pregnancy changed with the greatest abundance toward term pregnancy (quadratic effect of day, *P* < 0.05; Fig 1A). On Days 12 and 15 post-estrus, the expression of *CYP27B1* was not affected by day, status, or day × status, while steady-state levels of *CYP27B1* mRNA during pregnancy changed with the increased levels during mid- to late pregnancy (linear effect of day, *P* < 0.01; Fig 1B). Expression of *CYP24A1* mRNA on Days 12 and 15 post-estrus was affected by day (*P* < 0.05) and day × status interaction (*P* < 0.05) but not by pregnancy status and was greater on Day 15 of the estrous cycle than on Day 15 of pregnancy. Steady-state levels of *CYP24A1* mRNAs during pregnancy were highest on Days 12 and 15 and decreased thereafter (linear effect of day, *P* < 0.05; Fig 1C). Expression of *GC* mRNA was affected by day (*P* < 0.05), but not by pregnancy status and day × status interaction on Days 12 and 15 post-estrus. Steady-state levels of *GC* mRNAs changed during pregnancy with the highest abundance on Day 60 of pregnancy (quadratic effect of day, *P* < 0.01; Fig 1D). On days 12 and 15 post-estrus, expression of *VDR* was not affected by day, status and day × status, while steady-state levels of *VDR* mRNA during pregnancy changed with the greatest abundance being found on Day 90 of pregnancy (cubic effect of day, *P* < 0.01; Fig 1E).

### Localization of mRNAs for vitamin D hydroxylases, *CYP2R1*, *CYP24A1*, and *CYP27B1*, vitamin D binding protein, *DC*, and vitamin D receptor, *VDR*, and immunohistochemical localization of VDR protein in the endometrium during the estrous cycle and pregnancy in pigs

**Fig 2:**
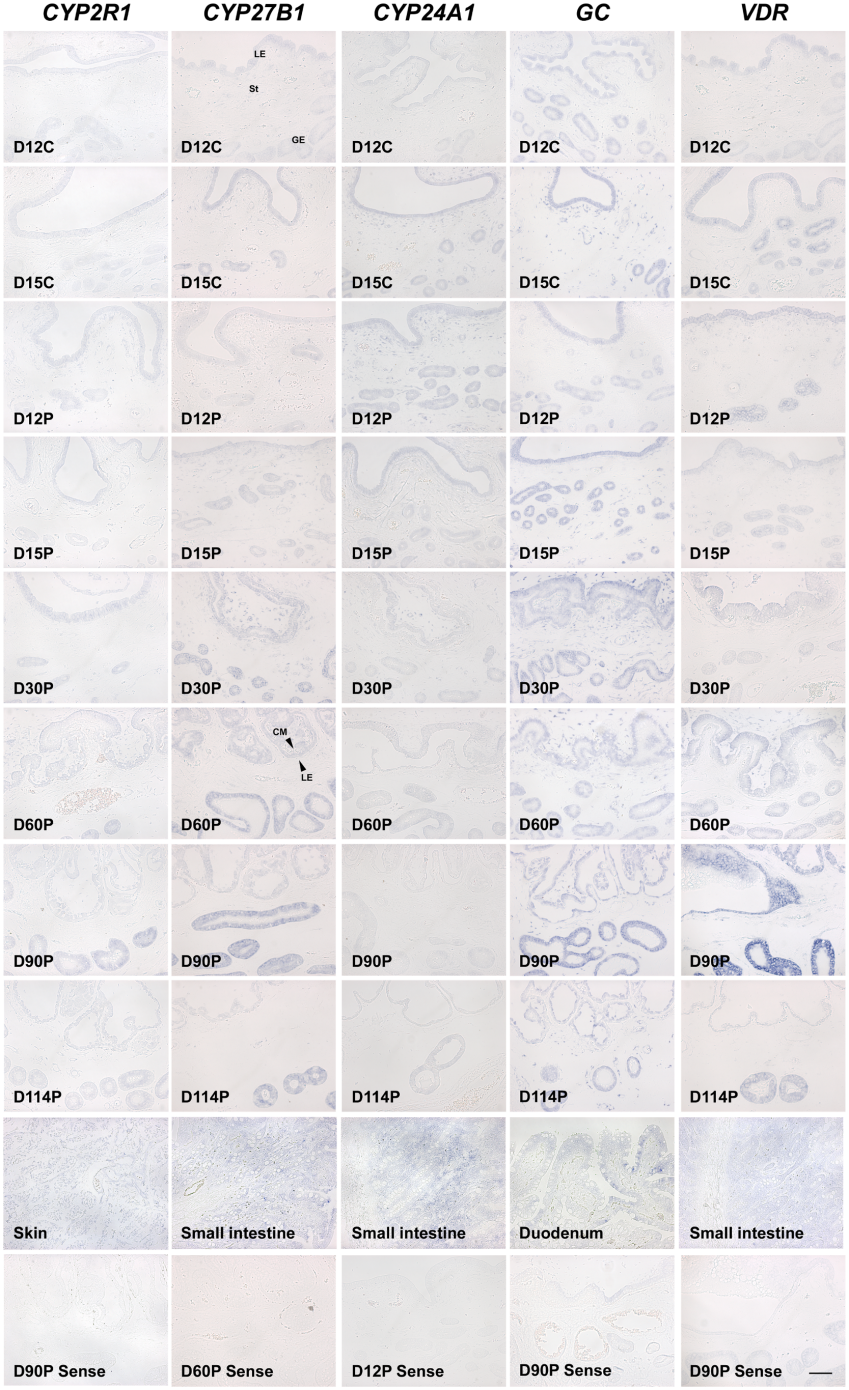
Localization of *CYP2R1*, *CYP27B1*, *CYP24A1*, *GC*, and *VDR* mRNAs in the endometrium during the estrous cycle and pregnancy by in situ hybridization analysis. Tissue sections from skin, small intestine, or duodenum hybridized with DIG-labeled anti-sense *CYP2R1*, *CYP27B1*, *CYP24A1*, *GC*, or *VDR* cDNA probes served as the positive control, and representative uterine sections from the indicated day of pregnancy hybridized with DIG-labeled sense *CYP2R1*, *CYP27B1*, *CYP24A1*, *GC*, or *VDR* cDNA probes (Sense) served as the negative control. D, Day; C, estrous cycle; P, pregnancy; LE, luminal epithelium; GE, glandular epithelium; St, stroma; CM, chorionic membrane. Scale bar = 100 μm.

To investigate the cellular localization of *CYP2R1*, *CYP27B1*, *CYP24A1*, *GC*, and *VDR* mRNAs in the endometrium during the estrous cycle and pregnancy in pigs, we performed *in situ* hybridization analysis. *CYP2R1*, *CYP27B1*, and *VDR* mRNAs were localized primarily to luminal (LE) and glandular epithelial (GE) cells in the endometrium during the estrous cycle and pregnancy (Fig 2). *CYP24A1* and *GC* mRNAs were detectable in LE, GE and stromal cells in the endometrium during the estrous cycle and pregnancy. During pregnancy, expression of *CYP2R1*, *CYP27B1*, *CYP24A1*, *GC*, and *VDR* mRNAs was also detected in the chorionic membrane.

**Fig 3:**
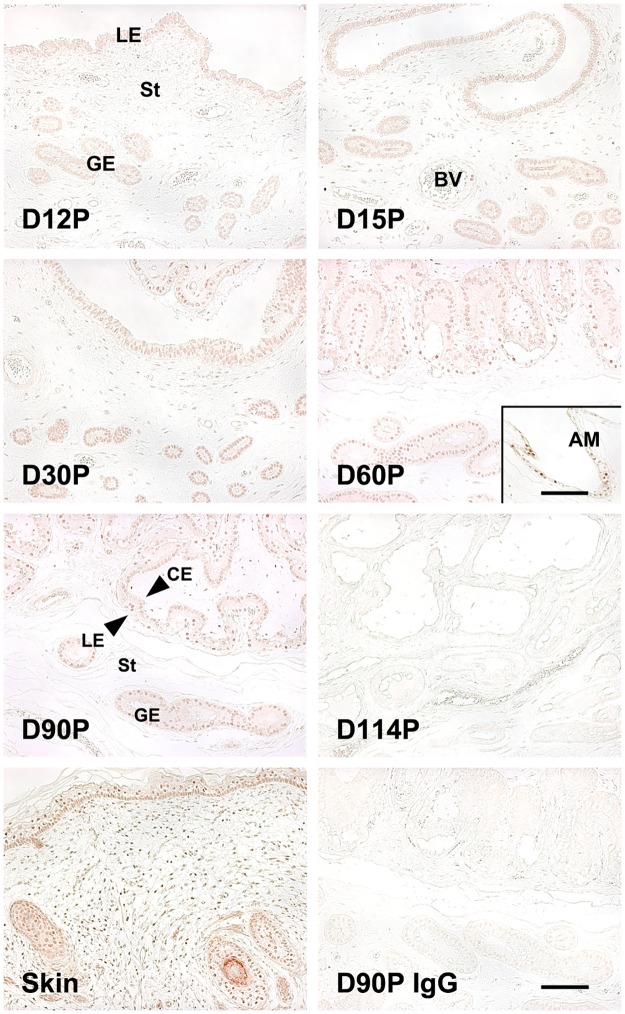
Immunohistochemical analysis of vitamin D receptor (VDR) protein in the endometrium during the estrous cycle and pregnancy in pigs. Immunoreactive VDR protein was detected in the endometrial epithelial and stromal cells during the estrous cycle and pregnancy and in chorioallantoic tissues during pregnancy. A representative uterine section from Day 90 of pregnancy immunostained with normal rabbit IgG as a negative control (D90P IgG) is shown. D, Day; C, estrous cycle; P, pregnancy; LE, luminal epithelium; GE, glandular epithelium; St, stroma; CM, chorionic epithelium; AM, allantoic membrane. Bars = 100 μm and 50 μm in inset.

To determine localization of the VDR protein in the endometrium we performed immunohistochemistry. The VDR protein was predominantly localized to the nucleus of the cells in the endometrium and was detected in the uterine LE, GE and stromal cells during the estrous cycle and pregnancy (Fig 3). The VDR protein was also detected in chorionic epithelial cells as well as in the allantoic membrane during mid- to late pregnancy.

### Expression of vitamin D_3_ hydroxylases, *CYP2R1*, *CYP24A1*, and *CYP27B1*, vitamin D binding protein, *DC*, and vitamin D receptor, *VDR*, in conceptuses on Day 12 and Day 15 of pregnancy and in chorioallantoic tissues during later stages of pregnancy

**Fig 4:**
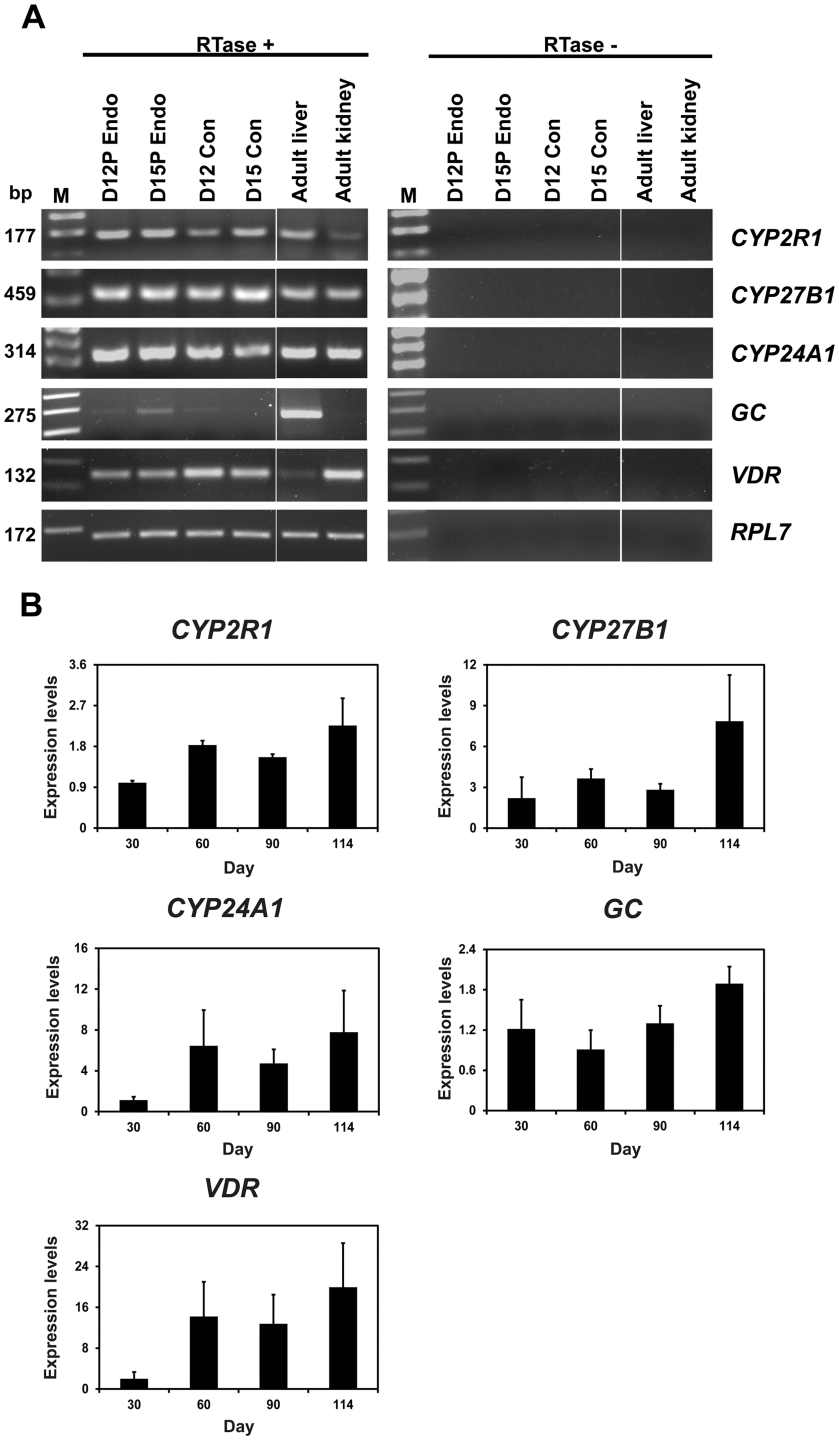
Expression of *CYP2R1*, *CYP27B1*, *CYP24A1*, *GC*, and *VDR* by conceptuses from Day 12 and Day 15 of pregnancy (A) and by chorioallantoic tissues during later stages of pregnancy (B). **A.** RT-PCR analysis of *CYP2R1*, *CYP27B1*, *CYP24A1*, *GC*, and *VDR* mRNA in conceptuses on Day 12 and Day 15 of pregnancy as well as in liver and kidney tissues as positive controls was performed using total RNA preparations. *RPL7* was used as a positive control. RTase +/-, with (+) or without (-) reverse transcriptase; M, molecular marker; D12 Endo, endometrium on Day 12 of pregnancy; D12 Con, Day 12 conceptus; D15 Con, Day 15 conceptus. **B**. Real-time RT-PCR analysis of the expression of *CYP2R1*, *CYP27B1*, *CYP24A1*, *GC*, and *VDR* mRNAs in chorioallantoic tissue samples on Days 30, 60, 90, and 114 of pregnancy. Data are reported as expression relative to that detected on Day 30 of pregnancy after normalization of the transcript amount to the endogenous *RPL7* control, and data are presented as mean with standard error.

To determine whether during early pregnancy the conceptuses expressed *CYP2R1*, *CYP27B1*, *CYP24A1*, *GC*, and *VDR* mRNAs, we performed RT-PCR using cDNA from Days 12 and 15 conceptuses. *CYP2R1*, *CYP27B1*, *CYP24A1*, *GC*, and *VDR* mRNAs were expressed by conceptuses during early pregnancy, as well as in the adult liver and/or kidney tissues that were used as positive controls (Fig 4A).

Real-time RT-PCR analysis was performed to determine whether the abundance of *CYP2R1*, *CYP27B1*, *CYP24A1*, *GC*, and *VDR* mRNAs changed in chorioallantoic tissues during mid- to late pregnancy. As shown in Fig 4B, the abundance of *CYP2R1* and *VDR* mRNAs in chorioallantoic tissues increased toward term pregnancy (linear effect of day, *P* < 0.05 for *CYP2R1*; *P* = 0.0526 for *VDR*), while the abundance of *CYP27B1*, *CYP24A1*, and *GC* mRNAs did not change in chorioallantoic tissues on Days 30, 60, 90, and 114 of pregnancy.

### Levels of calcitriol in blood plasma and endometrial tissues during pregnancy and uterine flushings on Days 12 and 15 of the estrous cycle and pregnancy in pigs

**Fig 5:**
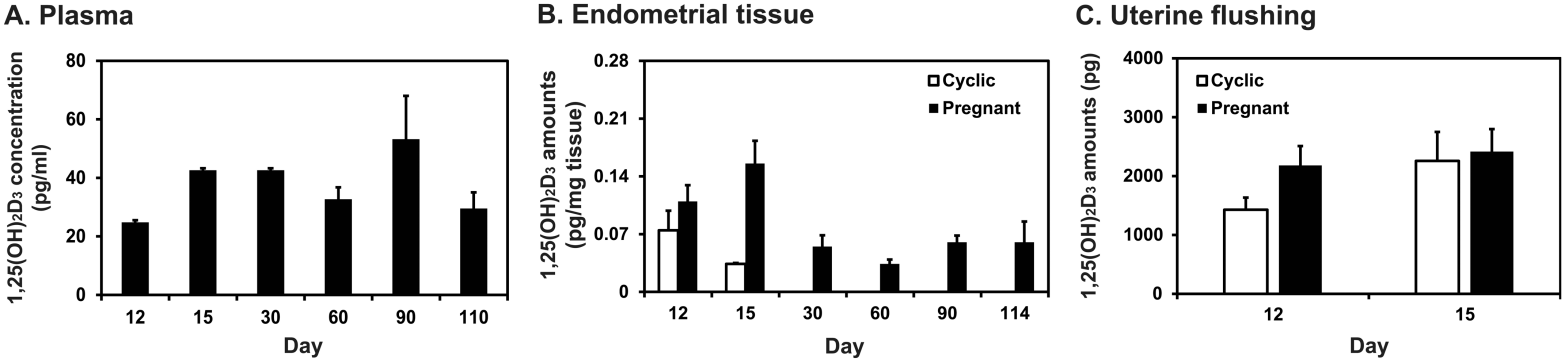
Levels of calcitriol [1,25(OH)_2_D_3_] in plasma blood during pregnancy (A), endometrial tissues (B), and uterine flushings (C) during the estrous cycle and pregnancy in pigs. Levels of calcitriol in plasma blood, endometrial tissue lysate, and uterine flushings were determined by ELISA. Calcitriol levels in the endometrial tissues were standardized per total tissue weight, and the amounts of calcitriol in the uterine flushings were total recoverable amounts of calcitriol. Data are presented as means with standard errors.

Having determined that vitamin D hydroxylases, *CYP2R1*, *CYP24A1*, and *CYP27B1*, vitamin D binding protein, *DC*, and vitamin D receptor, *VDR*, were expressed at the maternal-conceptus interface, the next experiment was performed to determine the levels of calcitriol in blood plasma and uterine endometrial tissues during pregnancy and in uterine flushings on Days 12 and 15 of the estrous cycle and pregnancy by ELISA. Concentration of calcitriol in plasma changed during pregnancy with the highest levels on Day 90 of pregnancy (quartic effect of day, *P* < 0.05; Fig 5A). In uterine endometrial tissues on Days 12 and 15 post-estrus, levels of calcitriol were affected by pregnancy status (*P* < 0.05) but not by day or day × status interaction. Concentration of calcitriol in endometrial tissues during pregnancy was highest on Days 12 and 15 and decreased thereafter (linear effect of day, *P* < 0.05) (Fig 5B). In uterine flushings on Days 12 and 15 post-estrus, total recoverable amounts of calcitriol were not affected by day, pregnancy status, or day × status interaction (Fig 5C).

### Effects of calcitriol on endometrial gene expression in the uterine endometrial tissues from Day 12 of the estrous cycle

Since that vitamin D hydroxylases, *CYP2R1*, *CYP24A1*, and *CYP27B1*, vitamin D binding protein, *DC*, and vitamin D receptor, *VDR*, were expressed in the endometrium, and levels of calcitriol in the endometrial tissues were highest on Days 12 and 15 during pregnancy, we hypothesized that endometrial calcitriol might affect the expression of genes involved in conceptus implantation, vitamin D metabolism, calcium ion regulation, PG metabolism and transport, and calcium-binding proteins in the porcine endometrium. Thus, we chose genes well known for these categories: 1) *FGF7*, *LPAR3*, *SPP1*, and *STC1* for conceptus implantation, 2) *CYP2R1*, *CYP27B1*, *CYP24A1*, and *VDR* for vitamin D metabolism, 3) *ATP2B1* (ATPase plasma membrane Ca^2+^ transporting 1), *S100G*, *SLC8A1* (solute carrier family 8 member A1), and *TRPV6* for calcium ion regulation [10, 37], 4) *ABCC4* (ATP-binding cassette sub-family C member 4), *AKR1B1*, *PTGS1* (prostaglandin-endoperoxide synthase 1), and *PTGS2* for PG-related molecules [3, 38], and 5) *S100A7A*, *S100A8*, *S100A9*, and *S100A12* for calcium binding proteins [39, 40]. We determined the effect of calcitriol on the expression of these genes in uterine endometrial explant tissues from Day 12 of the estrous cycle.

**Fig 6:**
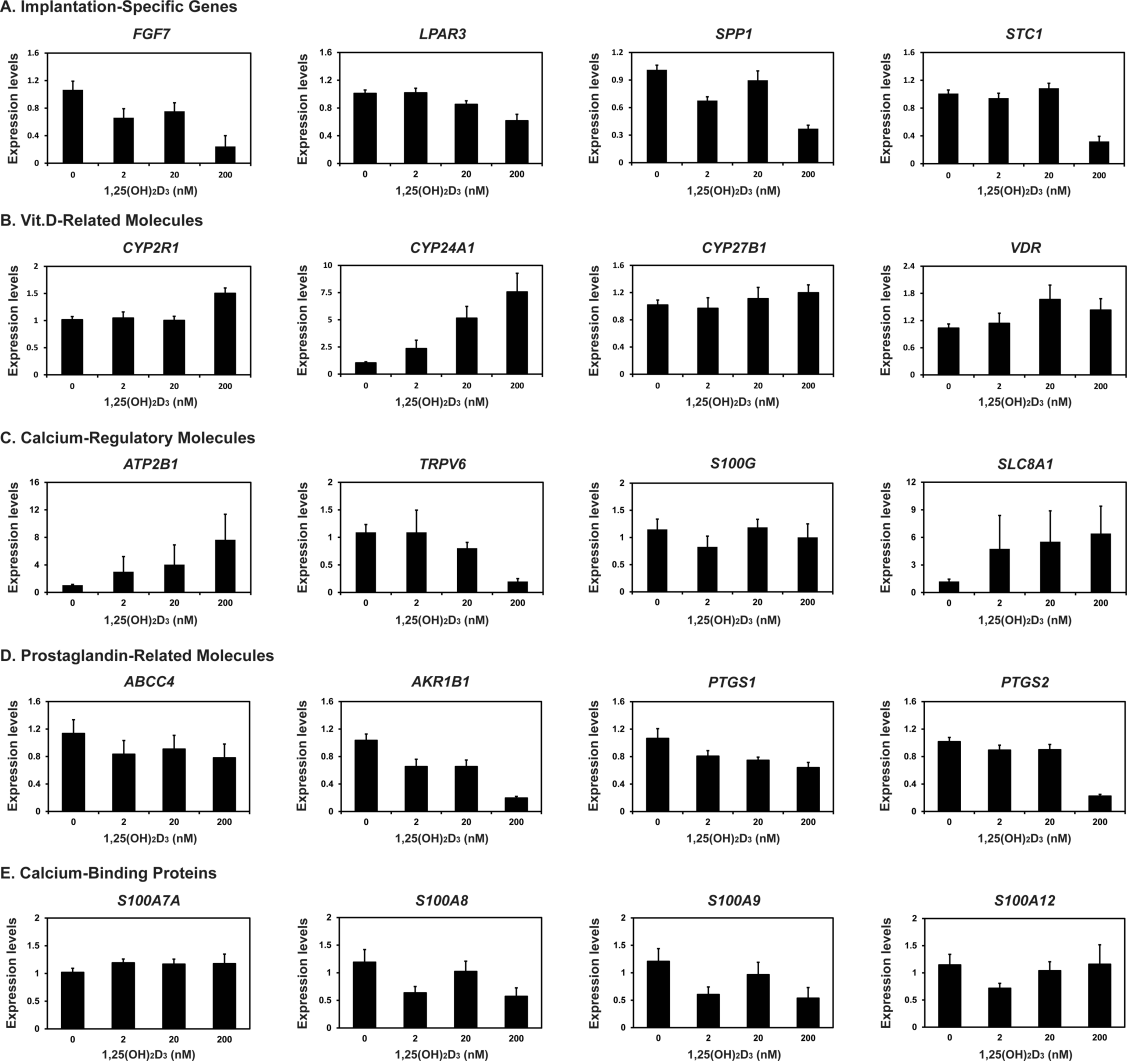
Effects of calcitriol [1,25(OH)_2_D_3_] on the expression of implantation-related genes (A), vitamin D-related genes (B), calcium-regulatory genes (C), prostaglandin-related genes (D), and calcium-binding protein genes (E) in endometrial tissue explants. Endometrial explants from gilts on Day 12 of the estrous cycle were cultured in the presence of 0, 2, 20, and 200 μM calcitriol. Levels of mRNAs for each endometrial gene were analyzed by real-time RT-PCR, and abundance of mRNAs is presented as expression relative to that for mRNAs in the control group of endometrial explants (0 ng/ml calcitriol) after normalization of transcript amounts to *RPL7* mRNA. Data are presented as means with standard errors. For each treatment, all experiments were repeated in triplicate using endometrial tissue from each of three gilts.

The expression of the implantation-related molecules *FGF7*, *LPAR3*, *SPP1*, and *STC1* mRNAs was decreased by increasing doses of calcitriol (linear effect of dose, *P* < 0.01 for all molecules) (Fig 6A). The expression of *CYP2R1*, *CYP24A1*, and *VDR* mRNAs was increased with increasing doses of calcitriol (linear effect of dose, *P* < 0.01 for *CYP2R1* and *CYP24A1*; quadratic effect of dose, *P* = 0.059 for *VDR*), while the expression of *CYP27B1* mRNA was not affected by calcitriol (Fig 6B). In addition, among the calcium-regulatory molecules investigated, the expression of *TRPV6* mRNAs was decreased with increasing doses of calcitriol (linear effect of dose, *P* < 0.01), but the expression of *S100G*, *ATP2B1*, and *SLC8A1* mRNAs was not affect by calcitriol (Fig 6C). For PG-related molecules, calcitriol decreased the expression of *AKR1B1*, *PTGS1*, and *PTGS2* but not of *ABCC4* (linear effect of dose, *P* < 0.01 for *AKR1B1* and *PTGS2*; *P* < 0.05 for *PTGS1*) (Fig 6D). The expression of calcium binding proteins *S100A8* and *S100A9* mRNAs, but not that of *S100A7A* and *S100A12* mRNAs, was decreased with increasing doses of calcitriol (cubic effect of dose, *P* < 0.05 for *S100A8* and *S100A9*) (Fig 6E).

## Discussion

The significant findings of this study are: 1) vitamin D-metabolizing enzymes *CYP2R1*, *CYP27B1*, and *CYP24A1*, vitamin D binding protein, *GC*, and vitamin D receptor, *VDR* are expressed in the endometrium in a pregnancy status- and/or stage-specific manner; 2) *CYP2R1*, *CYP27B1*, *CYP24A1*, *GC*, and *VDR* mRNAs are expressed in conceptuses on Days 12 and 15 of pregnancy and in chorioallantoic tissues from Day 30 to term pregnancy; 3) levels of calcitriol in the uterine endometrial tissues are higher on Days 12 and 15 of pregnancy than in the later stage of pregnancy, while calcitriol levels in plasma are high during late pregnancy; and 4) calcitriol affects the expression of several genes related to conceptus implantation, vitamin D metabolism, calcium ion regulation, PG metabolism, and calcium-binding proteins. To the best of our knowledge, this is the first report characterizing the expression profiles of *CYP2R1*, *CYP27B1*, *CYP24A1*, *GC*, and *VDR* and the role of calcitriol at the maternal-conceptus interface during pregnancy in pigs.

It has been reported that calcitriol is produced not only by kidney but also by the uterus during the reproductive cycle and pregnancy, and the enzymes involved in calcitriol metabolism are expressed by the endometrium in humans and mice [24, 41–43]. In this study, we found that the genes encoding vitamin D-metabolizing enzymes, *CYP2R1*, *CYP27B1*, and *CYP24A1*, a vitamin D binding protein, *GC*, and a receptor for vitamin D, *VDR*, are also expressed at the mRNA level in the endometrium during the estrous cycle and pregnancy in pigs. Furthermore, we found that abundance of *CYP2R1*, *CYP27B1*, *CYP24A1*, *GC*, and *VDR* expression in the endometrium during pregnancy changed in a stage-specific fashion; abundance of *CYP2R1* mRNAs was greater in late pregnancy than in early and mid-pregnancy; abundance of *CYP27B1*, *GC*, and *VDR* mRNAs was greater during mid- to late pregnancy than during early pregnancy; and abundance of *CYP24A1* mRNAs was greater during early pregnancy than during mid- to late pregnancy. These results indicate that expression of vitamin D metabolizing enzyme and related molecules in the endometrium is dynamically regulated during pregnancy in pigs. Additionally, the finding that the expression of endometrial *CYP24A1* mRNA is greater in the early stage of pregnancy than in mid- to late pregnancy suggests that high metabolic activity may be present during early pregnancy to catabolize calcitriol in the endometrium. Indeed, the levels of calcitriol in endometrial tissues during pregnancy were higher on Days 12 and 15 than in the later stage of pregnancy. It has been shown in mice that the promoter region of the *Cyp24a1* gene contains a vitamin D response element and is responsible for calcitriol-induced expression of *Cyp24a1* [44]. Our current results also showed that *CYP24A1* expression was increased by calcitriol in endometrial explant tissues. Thus, it is likely that the increased endometrial expression of *CYP24A1* is caused by the increased calcitriol levels in the endometrium on Days 12 and 15 of pregnancy, and that it forms a local negative feedback mechanism to regulate calcitriol concentrations in the endometrium.

*CYP2R1*, *CYP27B1*, *CYP24A1*, *GC*, and *VDR* are expressed in the human placenta [23, 25, 45], and *CYP24A1* and *VDR* are expressed in the mouse placenta [26, 27]. In this study of pigs, we found that *CYP2R1*, *CYP27B1*, *CYP24A1*, *GC*, and *VDR* were expressed by early stage (Days 12 and 15) conceptuses and by chorioallantoic tissues during the later stage pregnancy, suggesting that the expression of *CYP2R1*, *CYP27B1*, *CYP24A1*, *GC*, and *VDR* in the placenta is a common feature among species with different placentation types. The expression of *CYP2R1* and *VDR* mRNAs in chorioallantoic tissues increased toward term pregnancy in pigs. In mice, placental VDR protein levels are higher during late pregnancy than mid-pregnancy [27]. Since calcitriol increases the expression of *CYP2R1* and *VDR* in endometrial explant tissues (this study) and in human endometrial cancer [24] and oral squamous carcinoma cells [46], it is likely that calcitriol at the maternal-conceptus interface may affect the expression of *CYP2R1* and *VDR* in chorioallantoic tissues. In addition, the expression of *CYP2R1* is induced by IL-1β in human endometrial stromal cells [41], and the expression of *CYP2R1* and *VDR* is increased by the combination of tumor necrosis factor-α and IFNG in human brain pericytes [47], suggesting that some cytokines may also be involved in regulation of *CYP2R1* and *VDR* expression.

The results of this study showed that *CYP2R1*, *CYP27B1*, *CYP24B1*, *GC*, and *VDR* mRNAs were localized to endometrial epithelial and stromal cells with differential expression depending the cell types and stage in the endometrium during the estrous cycle and pregnancy. This is similar to the findings that CYP2R1, CYP27B1, CYP24A1, and VDR proteins are localized to both epithelial and stromal cells with varying amounts depending on the menstrual cycle in humans [24, 41]. The VDR protein is localized to LE, GE, and stromal cells in the endometrium, with higher levels during the estrus phase than in other phases of the estrous cycle in mice [43]. Our results also showed that VDR proteins were localized to the endometrial epithelial and stromal cells during the estrous cycle and during pregnancy in pigs. In addition, we found that VDR proteins were detectable in chorionic and allantoic epithelial cells during pregnancy in pigs.

Since the vitamin D-metabolizing enzymes *CYP2R1*, *CYP27B1*, and *CYP24A1* mRNAs were expressed in the endometrium with greater abundance of *CYP2R1* and *CYP27B1* mRNAs and lower abundance of *CYP24A1* mRNAs in late pregnancy than in early pregnancy, we postulated that endometrial calcitriol levels might change during pregnancy with increasing amounts toward term pregnancy. We found that calcitriol concentrations in the endometrium during pregnancy did change but with higher levels on Days 12 and 15 than in the later stage of pregnancy. Maternal plasma calcitriol concentrations during pregnancy changed with the highest levels on Day 90 of pregnancy. The reason that endometrial calcitriol concentrations during late pregnancy were lower than early pregnancy is not yet clear, but it is possible that protein amounts or enzymatic activity for CYP2R1, CYP27B1, and CYP24A1 in the endometrium during late pregnancy may be regulated by other mechanism(s) to control endometrial calcitriol metabolism. It is likely that the increased systemic production of calcitriol due to the maternal calcium requirement during late pregnancy negates the necessity of local calcitriol production in the endometrium. In addition, since we did not measure the protein amounts and enzymatic activities of *CYP2R1*, *CYP27B1*, and *CYP24B1* in the endometrium during pregnancy, mRNA levels may not be reflective of protein levels, and protein levels may not be reflective of active enzymatic activity, it may be caused by the differential regulation of protein expression in the endometrium during pregnancy. Nevertheless, these results suggest that the concentration of calcitriol in the local microenvironment of the endometrium may be regulated tightly and differently to systemic calcitriol concentrations, and enzymes locally expressed in the endometrial tissues may be involved in that process. The finding that concentrations of calcitriol in maternal plasma were highest during late pregnancy was similar to the report in humans that plasma levels of calcitriol during pregnancy are higher than in non-pregnancy and that these levels gradually increase from early to late stage of pregnancy [48]. The changes in calcitriol concentrations may be related to a physiological response to increased calcium requirements during the late stage of pregnancy. Indeed, total calcium amounts in endometrial tissues during pregnancy are at their highest levels during mid- to late pregnancy in pigs (Choi and Ka, unpublished data).

Because endometrial calcitriol concentrations were higher on Days 12 and 15 of pregnancy than in the later stage of pregnancy, we hypothesized that calcitriol may be involved in endometrial gene expression during early pregnancy. Thus, we investigated the effect of calcitriol on the expression of some genes known to be involved in conceptus implantation (*FGF7*, *LPAR3*, *SPP1*, and *STC1*), vitamin D metabolism (*CYP2R1*, *CYP27B1*, *CYP24A1*, and *VDR*), calcium ion regulation (*TRPV6*, *S100G*, *ATP2B1*, and *SLC8A1*), PG metabolism and transport (*ABCC4*, *AKR1B1*, *PTGS1*, and *PTGS2*), and calcium-binding proteins (*S100A7A*, *S100A8*, *S100A9*, and *S100A12*). Indeed, in the endometrial explant culture study we found that the expression of several endometrial genes was affected by increasing doses of calcitriol; calcitriol increased the expression of *CYP2R1*, *CYP24A1*, and *VDR* mRNAs but decreased the expression of *AKR1B1*, *FGF7*, *LPAR3*, *PTGS1*, *PTGS2*, *SPP1*, *STC1*, *TRPV6*, *S100A8*, and *S100A9* mRNAs in endometrial explants. The expression of *ABCC4*, *ATP2B1*, *CYP27B1*, *S100A7A*, *S100A12*, *S100G*, and *SLC8A1* mRNAs was not affected by calcitriol. These results suggest that calcitriol regulates the expression of endometrial genes related to conceptus implantation, vitamin D metabolism, calcium ion regulation, PG metabolism, and calcium-binding proteins in the porcine endometrium.

In pigs, endometrial expression of *AKR1B1*, *FGF7*, *LPAR3*, *SPP1*, *STC1*, *S100G*, and *TRPV6* mRNAs during early pregnancy is induced by estrogen [3–5, 7, 9, 10, 49], and our preliminary results showed that *S100A8* and *S100A9* mRNAs are also up-regulated by estrogen in endometrial tissues in pigs (Choi and Ka, unpublished data). Especially, the expression of *AKR1B1*, *FGF7*, *LPAR3*, *SPP1*, and *STC1* mRNAs markedly increase in endometrial LE cells in response to conceptus-derived estrogen on Days 12 and 15 and decrease thereafter in LE cells [3–5, 9, 49], although the expression of *FGF7* and *SPP1* mRNAs increase in GE cells during mid- to late pregnancy [4, 9, 49]. FGF7 and LPAR3 are involved in proliferation and differentiation of conceptus trophectoderm and LPAR3 and AKR1B1 act on endometrial PG synthesis [2, 4, 5]. SPP1 is an extracellular matrix protein and plays a critical role in cell-to-cell adhesion between the trophectoderm cells and endometrial epithelial cells during the implantation period in pigs [2, 49]. Thus, the result that calcitriol decreased the endometrial expression of *AKR1B1*, *FGF7*, *LPAR3*, *SPP1*, *STC1*, *TRPV6*, *S100A8*, and *S100A9* mRNAs in this study suggests that calcitriol may counteract the action of estrogen in endometrial LE cells during the peri-implantation period in pigs. In addition, since estrogen increases endometrial secretion of calcium into the uterine lumen at the time of conceptus implantation [11], and the major function of calcitriol is to increase blood calcium concentrations [14], it is feasible that estrogen and calcitriol cooperate in endometrial calcium secretions. However, the detailed mechanism regarding how estrogen and calcitriol interact to regulate endometrial gene expression and calcium secretion is not currently known and needs further study.

In addition to the role on calcium homeostasis, vitamin D plays important roles in the regulation of immune response by targeting various immune cells, including monocytes, macrophages, dendritic cells, and T and B lymphocytes [34]. Vitamin D enhances the antimicrobial properties of monocytes and macrophages and modulates cytokine production in antigen presenting cells and T lymphocytes by increasing Th2 cytokines, IL4, IL10, and TGF-β, and inhibiting the Th1 cytokines, IFNG, IL2, IL12, and IL23 [34]. It has been shown that vitamin D suppresses the expression of IFNG in decidual natural killer (NK) cells [50], affects decidual dendritic cells and macrophages to activate regulatory T cells [33], and increases secretion of Th2 cytokines, while inhibiting release of Th1 cytokines at the maternal-fetal interface in humans [18]. In humans, it is well established that Th2 cytokines increase at the maternal-fetal interface and the imbalance between Th1 and Th2 cytokines causes complicated pregnancy problems, such as recurrent pregnancy loss, implantation failure, preterm labor, and preeclampsia [51, 52]. Th1 cytokines activate cell-mediated immunity that is deleterious for pregnancy, while Th2 cytokines are involved in humoral immunity that is beneficial for fetal survival and leads to maternal immune tolerance to the developing fetus [52]. In the porcine endometrium, there are many immune cells recruited into the endometrium during early pregnancy [53] and recruitment of T cells and NK cells into the endometrium is greatest on Day 15 of pregnancy [54], when the conceptus attachment to the endometrial epithelial cells occurs. Our results indicate that IL10 production increases and IL12 production decreases in the endometrium during early pregnancy (Han and Ka, unpublished data). Thus, it is possible that calcitriol plays a role in regulation of immune cell activity and Th2 cytokine production to modulate maternal immunity during the implantation period in pigs, although this needs further analysis.

It has been shown that there is a novel pathway for vitamin D metabolism generating vitamin D-hydroxyderivatives, including 20-hydroxyvitamin D, 22-hydroxyvitamin D,20,22-dihydroxyvitamin D, 20,23-dihydroxyvitamin D, and 1,20-dihydroxyvitamin D, in placenta, adrenal gland, and epidermis in humans [55–58]. This novel pathway for vitamin D metabolism is mediated by CYP11A1, which catalyzes the conversion of cholesterol to pregnenolone in steroidogenesis, and CYP27B1 [58]. Although it was not determined in this study, the expression of CYL11A1 and function of novel vitamin D metabolites at the maternal-fetal interface also need to be investigated.

In conclusion, our results indicate that the vitamin D-metabolizing enzymes, *CYP2R1*, *CYP27B1*, and *CYP24A1*, vitamin D binding protein, *GC*, and vitamin D receptor, *VDR*, are expressed in the endometrium, early stage conceptuses and placental tissues during pregnancy with differential expression profiles, and endometrial calcitriol levels are higher during early pregnancy than in later stage pregnancy. Calcitriol affects the expression of endometrial genes related to conceptus implantation, vitamin D metabolism, calcium ion regulation, PG metabolism, and calcium-binding proteins. These results provide important insights into the metabolism of vitamin D at the maternal-fetal interface and the function of vitamin D in endometrial gene expression at the time of implantation in pigs.

## Supporting information

S1 Table Summary of primer sequences and expected product sizes. (DOCX)
