# Supplementary material for: Vitamin D-metabolic enzymes and related molecules: Expression at the maternal-conceptus interface and the role of vitamin D in endometrial gene expression in pigs
Source: PLoS One. 2017 Oct 31;12(10):e0187221. doi: 10.1371/journal.pone.0187221 (PMC5663432; doi:10.1371/journal.pone.0187221)
Supplement: S1 Table — (DOCX) [file pone.0187221.s001.docx]

**Table 1.** Summary of primer sequences and expected product sizes

| **Gene Name** | **Primer Sequences (5’ 🡪 3’)** | **Product Size (bp)** | **GenBank Accession No.** |
| --- | --- | --- | --- |
| RT-PCR |  |  |  |
| *CYP2R1* | F:5’-CTGAAACTACAACCAATGTGTTACG-3’ | 177 | KP687259.1 |
|  | R:5’-ATAAGTGAATCGTTCTCCAAAAATG-3’ |  |  |
| *CYP27B1* | F:5’-TACATTATCCCCAAAAATACACTGG-3’ | 316 | NM_213995.1 |
|  | R:5’-ACTGTAGATTGATGCTTCTCTCAGG-3’ |  |  |
| *CYP24A1* | F:5’-TTCAAGTCAGTCAAATCTTGTATCG-3’ | 314 | NM_214075.2 |
|  | R:5’-TCTTTCAGACAGGCTTTTAAATACG-3’ |  |  |
| *GC* | F:5’-TCCTCAGTAAAATACTTGAGCCAAC-3’ | 275 | XM_003356971.3 |
|  | R:5’-CTTGGAGGCAAAGTCTGAGC-3’ |  |  |
| *VDR* | F:5’-AGGCTTCTTCAGACGGAGCATGAA-3’ | 143 | EF405627.1 |
|  | R:5’-ACTCCTTCATCATGCCGATGTCCA-3’ |  |  |
| *RPL7* | F:5’-AAGCCAAGCACTATCACAAGGAATACA-3’ | 172 | NM_001113217 |
|  | R:5’-TGCAACACCTTTCTGACCTTTGG-3’ |  |  |
| In Situ Hybridization |  |  |  |
| *CYP2R1* | F:5’-TTAGAAGAAACCAAATTTTTCATGG-3’ | 132 | KP687259.1 |
|  | R:5’-ATAAGTGAATCGTTCTCCAAAAATG-3’ |  |  |
| *CYP27B1* | F-5’-AATGACCATAGCAAAGTACTTGAGG-3’ | 459 | NM_213995.1 |
|  | R-5’-GTCAGTAGTTGAATTCTTCCTCAGC-3’ |  |  |
| *CYP24A1* | F:5’-TTCAAGTCAGTCAAATCTTGTATCG-3’ | 314 | NM_214075.2 |
|  | R:5’-TCTTTCAGACAGGCTTTTAAATACG-3’ |  |  |
| *GC* | F:5’-TATGAGAAGGATAAAGTCTGCAAGG-3’ | 1287 | XM_003356971.3 |
|  | R:5’-CTTGGAGGCAAAGTCTGAGC-3’ |  |  |
| *VDR* | F:5’-AGGCTTCTTCAGACGGAGCATGAA-3’ | 143 | EF405627.1 |
|  | R:5’-ACTCCTTCATCATGCCGATGTCCA-3’ |  |  |
| Real-Time PCR |  |  |  |
| **Implantation-Specific Genes** |  |  |  |
| *FGF7* | F: 5’-TACTATAATGCGCAAATGGATACTG-3’ | 209 | AF217463.1 |
|  | R: 5’-TTCTCACTCTTATATCCCCTCCTTC-3’ |  |  |
| *HOXA10* | F: 5’-AAAGAGCGGCCGGAAGAA-3’ | 120 | JN836600.1 |
|  | R: 5’-ACGCTGCGGCTGATCTCTAG-3’ |  |  |
| *LPAR3* | F: 5’-GCCGGAGGACACCCATGAAG-3’ | 111 | NM_001162402.1 |
|  | R: 5’-TGCAGTTCAGGCCGTCCAGT-3’ |  |  |
| *SPP1* | F: 5’-TTGGACAGCCAAGAGAAGGA-3’ | 121 | NM_214023.1 |
|  | R: 5’-GCTCATTGCTCCCATCATAGGTCTTG-3’ |  |  |
| **Vit.D-Related Molecules** |  |  |  |
| *CYP2R1* | F:5’-TTAGAAGAAACCAAATTTTTCATGG-3’ | 132 | KP687259.1 |
|  | R:5’-ATAAGTGAATCGTTCTCCAAAAATG-3’ |  |  |
| *CYP24A1* | F:5’-TTCAAGTCAGTCAAATCTTGTATCG-3’ | 314 | NM_214075.2 |
|  | R:5’-TCTTTCAGACAGGCTTTTAAATACG-3’ |  |  |
| *CYP27B1* | F-5’-AATGACCATAGCAAAGTACTTGAGG-3’ | 459 | NM_213995.1 |
|  | R-5’-GTCAGTAGTTGAATTCTTCCTCAGC-3’ |  |  |
| *VDR* | F:5’-AGGCTTCTTCAGACGGAGCATGAA-3’ | 143 | EF405627.1 |
|  | R:5’-ACTCCTTCATCATGCCGATGTCCA-3’ |  |  |
| *GC* | F:5’-TCCTCAGTAAAATACTTGAGCCAAC-3’ | 275 | XM_003356971.3 |
|  | R:5’-CTTGGAGGCAAAGTCTGAGC-3’ |  |  |
| **Calcium-Regulatory Molecules** |  |  |  |
| *ATP2B1* | F: 5’-TCGGTTGCCTATGGTGGAGT-3’ | 146 | X53456.1 |
|  | R: 5’-ACGTCGCCGTAGCTTTCCTG-3’ |  |  |
| *S100G* | F: 5’-TGAAAGGTCCGAGAACCCTAGATG-3’ | 141 | NM_214140.2 |
|  | R: 5’-TCCTTAAAATGGGACTGTTGCGTG-3’ |  |  |
| *STC1* | F: 5’-GTCAAAGAGAGTTTAAAGTGCATCG-3’ | 372 | NM_001103212.1 |
|  | R: 5’-ACGTTTTCTGTTGAAGTCAGCTC-3’ |  |  |
| *TRPV6* | F: 5’-CACTTTAGGAGAGGCTTGCTG-3’ | 147 | XM_003134594.1 |
|  | R: 5’-ATGACTTTATTGGAAGGTAGGGAGGT-3’ |  |  |
|  |  |  |  |
| **Prostaglandin-Related Molecules** |  |  |  |
| *ABCC4* | F: 5’-AAGATCACAATCTTAGTGACCCATC-3’ | 369 | XM_003357877 |
|  | R: 5’-ACCAGCTGTGAAGTAATCCTTGTAG-3’ |  |  |
| *AKR1B1* | F: 5’-ATGTGTACCAGAACGAGAACGAG-3’ | 192 | NM_001001539 |
|  | R: 5’-GGATAAGGTAGAGGTCCAGGTAGTC-3’ |  |  |
| *PTGS1* | F: 5’-CAACACTTCACCCACCAGTTCTTC-3’ | 99 | AF207823 |
|  | R: 5’-TCCATAAATGTGGCCGAGGTCTAC-3’ |  |  |
| *PTGS2* | F: 5’-TCGACCAGAGCAGAGAGATGAGAT-3’ | 134 | AF207824 |
|  | R: 5’-ACCATAGAGCGCTTCTAACTCTGC-3’ |  |  |
| **Calcium-Binding Proteins** |  |  |  |
| *S100A7A* | F: 5’-GCAGACAAGGACAAGGACAAC-3’ | 221 | ENSSSCG00000006591 |
|  | R: 5’-CTTGCCACAGACACACAAGG-3’ |  |  |
| *S100A8* | F: 5’-CTTGAAGAGATTGTTAGAGACGGAG-3’ | 171 | FJ263391.1 |
|  | R: 5’-TACTCTTTGTGGATGTCTTCATGG-3’ |  |  |
| *S100A9* | F: 5’-ATGCAGCATAGAAACCATTATCAAC-3’ | 256 | NM_001177906.1 |
|  | R: 5’-CATCTCCTCGTGAGAAGCTACC-3’ |  |  |
| *S100A12* | F: 5’-ATGACTAAGCTGGAAGACCATCTG-3’ | 254 | FJ263393.1 |
|  | R: 5’-GCAGTTATCAGCACATCAGTCAC-3’ |  |  |
| **Reference gene** |  |  |  |
| *RPL7* | F:5’-AAGCCAAGCACTATCACAAGGAATACA-3’ | 172 | NM_001113217 |
|  | R:5’-TGCAACACCTTTCTGACCTTTGG-3’ |  |  |
